# Demographic and Socioeconomic Risk Factors for Pain Progression and Recurrence in Middle-Aged and Older Adults

**DOI:** 10.1093/geroni/igaf122.1654

**Published:** 2025-12-31

**Authors:** Mikaela Bloomberg, Andrew Steptoe

**Affiliations:** University College London, London, England, United Kingdom; University College London, London, England, United Kingdom

## Abstract

Chronic pain is a major contributor to disability and reduced quality of life, particularly for older adults. Many pain conditions are also intermittent or progressive, with repeated episodes of pain worsening, subsiding, and recurrence. Identifying the demographic and socioeconomic factors that put individuals at risk of pain worsening or recurrence is therefore crucial for designing targeted early interventions and addressing disparities in pain management. Even so, the current evidence base is mixed and mostly uses methods that do not allow for nonlinear trajectories of pain. In this study, we used multistate models to examine demographic and socioeconomic risk factors for transitioning between pain states (moderate-severe, mild, or no pain) in 9,369 adults aged 50-98 years from the English Longitudinal Study of Ageing (study years: 2002/03-2021/23). This approach allowed us to capture the dynamic and recurrent nature of long-term pain. Findings highlighted the importance of gender and socioeconomic position: for women (compared with men) pain was less likely to improve in severity (hazard ratio [HR]=0.84, 95% confidence interval=0.74-0.96) and more likely to recur (HR = 1.45, 1.25-1.68). Higher education level was associated with pain improvement (HR = 1.43, 1.16-1.76), and lower risk of pain worsening (HR = 0.52, 0.42-0.64) and recurrence (HR = 0.67, 0.52-0.85); similar patterns were observed for wealth. These results emphasise the variability of long-term pain over time and support policies aimed at improving access to pain management for women and individuals with fewer socioeconomic resources. The findings also point to the importance of systematic long-term follow-up of vulnerable groups to enable timely intervention.

